# Serum Endocan Levels and Subclinical Atherosclerosis in Patients with Chronic Kidney and End-Stage Renal Diseases

**DOI:** 10.1155/2022/4524637

**Published:** 2022-07-13

**Authors:** Fatma M. El-Senosy, Rasha Elsayed Mohamed Abd El Aziz, Sammar Ahmed Kasim, Lamia Abdulbary Gad, Donia Ahmed Hassan, Seham Sabry, Ismail Mohamed El mancy, Taiseer Ahmed Shawky, Ibrahim Ghounim Ramadan Mohamed, Rady Elmonier, Essam Kotb, Abeer Mohammed Abdul-Mohymen

**Affiliations:** ^1^Department of Internal Medicine, Faculty of Medicine (Girls) Al-Azhar University, Cairo, Egypt; ^2^Department of Biochemistry, Faculty of Medicine Al Azhar University, Faculty of Medicine (Girls) Al-Azhar University, Cairo, Egypt; ^3^Department of Clinical Pathology, Faculty of Medicine (Girls) Al-Azhar University, Cairo, Egypt; ^4^Department of Internal Medicine, Faculty of Medicine, Al-Azhar University, Cairo, Egypt; ^5^Department of Internal Medicine, Faculty of Medicine, October 6 University, 6th of October, Egypt

## Abstract

**Background and Aim:**

Chronic kidney disease (CKD) and its final stage: end-stage renal disease (ESRD), are common clinical conditions. Endocan is a human endothelial cell-specific molecule produced by endothelial cells. Its production is related to activation of endothelium and angiogenesis. In this study, we assessed the relation between serum endocan levels and subclinical atherosclerosis (SCA) in CKD and hemodialysis (HD) patients.

**Subjects and Methods:**

The present case control study enrolled 30 patients on regular HD for at least 6 months, 30 patients with CKD, and 30 age and sex-matched healthy controls. All participants were subjected to careful history taking and thorough clinical examination. Laboratory investigations included complete blood count, kidney functions, and serum cholesterol, triglycerides, calcium, phosphorus, albumin, PTH, hsCRP, and endocan levels.

**Results:**

HD and CKD groups had significantly higher endocan levels when compared with control group (median (IQR): 519.0 (202.3–742.0) versus 409.0 (245.3–505.3) and 273.0 (168.0–395.5) ng/L, respectively). Also, HD patients had significantly higher endocan levels when compared with CKD levels. HD patients had significantly higher carotid intima-media thickness (CIMT) when compared with CKD patients (median (IQR): 0.80 (0.80–0.90) versus 0.75 (0.73–0.75) mm, *p* < 0.001). HD patients had significantly higher frequency of SCA when compared with CKD patients (46.7% versus 13.3%, *p*=0.005). Patients with SCA had significantly higher hsCRP (median (IQR): 36.5 (26.8–43.5) versus 24.0 (15.8–29.0) mg/dl) and endocan levels (697.0 (528.3–974.8) versus 222.5 (158.8–565.8) ng/L) when compared with patients without SCA. ROC curve analysis of endocan for identification of SCA in HD patients showed that at a cutoff of 380.5 ng/L, endocan has an AUC of 0.862 with a sensitivity and specificity of 92.9% and 68.7%, respectively.

**Conclusions:**

Serum endocan levels are related to SCA in HD patients. In addition, it is associated with the hyperinflammatory state in those patients.

## 1. Introduction

Chronic kidney disease (CKD) and its final stage: end-stage renal disease (ESRD), are common clinical conditions [1, 2]. CKD may be complicated by mineral and bone disorders, cardiovascular disease (CVD), and other complications [3]. Cardiovascular complications include coronary heart disease (CHD), congestive cardiac failure, and sudden arrest [4]. Atherosclerosis is one of the known cardiovascular complications in these patients [5]. Identification of subclinical atherosclerosis (SCA) in those patients is essential for prediction of forthcoming CVD [6].

Carotid intima-media thickness (CIMT) provides a non-invasive measurement of the intima and media thickening in carotid arteries as an early indicator of systemic atherosclerosis. It is correlated with CHD and stroke in the ESRD patients [7]. Early atherogenesis is associated with endothelial dysfunction [8]. Endocan is a human endothelial cell-specific molecule produced by endothelial cells. Its production is related to activation of endothelium and angiogenesis [9]. Elevation of serum endocan was previously linked to SCA in type 2 diabetic patients [10], systemic lupus erythematosus [11], and psoriasis [12].

In this study, we assessed the relation between serum endocan levels and SCA in CKD and HD patients.

## 2. Subjects and Methods

The present case control study was conducted at Al-Azhar University Hospitals, Al-Azhar University, Cairo, Egypt, from December 2020 to September 2021. The study protocol was approved by the ethical committee of Al-Azhar Faculty of Medicine, and all included subjects provided informed consent before enrollment in the study.

The study included 30 patients on regular HD for at least 6 months, 30 patients with chronic kidney disease (CKD) diagnosed according to National Kidney Foundation Disease Outcomes Quality Initiative (NKF-K/DOQI) clinical practice guidelines, and 30 age and sex-matched healthy controls. Patients were excluded if they had other causes of chronic inflammation, active malignancies, acute and chronic infection, kidney transplantation, or other cardiovascular diseases.

All participants were subjected to careful history taking and thorough clinical examination.

For laboratory assessment, 4 ml of peripheral venous blood was withdrawn from each individual and divided into two aliquots; 2 ml was collected in an EDTA tube for CBC. The remaining part was collected in serum separator tube, centrifuged at 3500 rpm for 10 min and divided into two parts; the first part was used for biochemical tests and for measurement of parathyroid hormone (PTH) and the remaining part of the serum was frozen at −20°C for analysis of endocan. Laboratory investigations included complete blood count, kidney functions, and serum cholesterol, triglycerides, calcium, phosphorus, albumin, and PTH levels. Determination of high-sensitivity C-reactive protein (hsCRP) was performed by commercial solid-phase immunosorbent assay (ELISA) kits (DRG International Inc., Springfield, New Jersey, USA). Measurement of serum endocan was performed using quantitative double-antibody sandwich ELISA kit (Bioassay Technology Laboratory, China, Cat. No. E3160Hu). CIMT measurement was done using B-mode ultrasound with a high definition L12-5 linear wideband probe (Philips HDI 5000, Bothell, Washington, USA). Patients were categorized to have SCA if their CIMT measurement was ≥0.9 mm [13].

Data were computerized and analyzed using IBM SPSS software package version 20.0. (IBM Corp., Armonk, New York, USA). Quantitative data were described using mean and standard deviation (SD) or median and interquartile range (IQR) and were compared using *t*-test, Mann–Whitney *U* test, or Kruskal–Wallis test as appropriate. Qualitative data were presented in numbers and percentages and were compared using Fisher's exact test or chi-square test as appropriate. Correlation analysis was achieved using Spearman's correlation coefficient. *p* value less than 0.05 was considered statistically significant. Receiver operator characteristic (ROC) curve analysis was used to identify diagnostic value of the investigated marker. *p* value less than 0.05 was considered statistically significant.

## 3. Results

**Figure 1:**
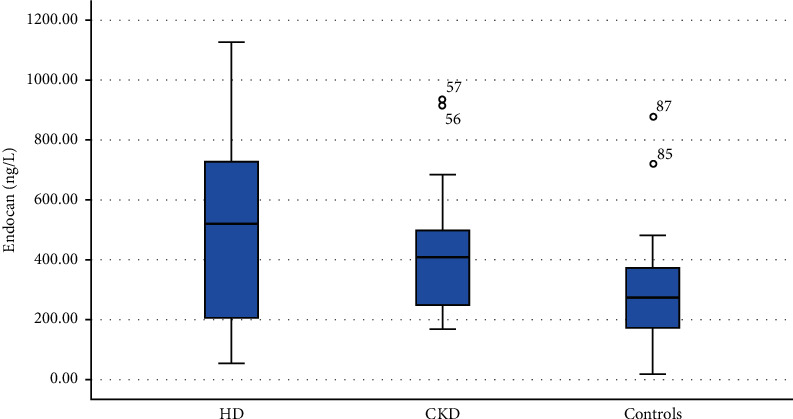
Serum endocan levels in the studied groups.

The present study included 30 patients under HD, 30 CKD patients, and 30 age and sex-matched healthy controls. HD and CKD groups had significantly higher endocan levels when compared with control group (median (IQR): 519.0 (202.3–742.0) versus 409.0 (245.3–505.3) and 273.0 (168.0–395.5) ng/L, respectively). Also, HD patients had significantly higher endocan levels when compared with CKD levels (Table 1, Figure 1).

Comparison between HD and CKD patients revealed significantly higher hsCRP levels in HD patients (median (IQR): 28.5 (21.8–39.5) versus 15.0 (9.8–20.3) mg/dl, *p* < 0.001). In addition, it was found that HD patients had significantly higher CIMT when compared with CKD patients (median (IQR): 0.80 (0.80–0.90) versus 0.75 (0.73–0.75) mm, *p* < 0.001). HD patients had significantly higher frequency of SCA when compared with CKD patients (46.7% versus 13.3%, *p*=0.005) (Table 1).

Comparison between HD patients with SCA and patients without showed that patients with SCA had significantly higher hsCRP (median (IQR): 36.5 (26.8–43.5) versus 24.0 (15.8–29.0) mg/dl) and endocan levels (697.0 (528.3–974.8) versus 222.5 (158.8–565.8) ng/L) when compared with patients without SCA (Table 2).

**Figure 2:**
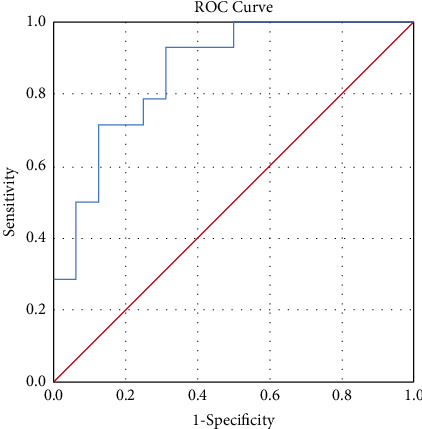
ROC curve for endocan levels in HD patients.

Correlation analysis identified significant correlation between endocan levels and PTH levels (*r* = 0.5, *p*=0.005), hsCRP levels (*r* = 0.55, *p*=0.002), and CIMT measurements (*r* = 0.59, *p*=0.001) (Table 3). ROC curve analysis of endocan for identification of SCA in HD patients showed that at a cutoff of 380.5 ng/L, endocan has an AUC of 0.862 with a sensitivity and specificity of 92.9% and 68.7%, respectively (Figure 2).

## 4. Discussion

Serum endocan levels showed significant changes in many acute and chronic conditions [14, 15]. The present study found significantly higher endocan levels in patients' groups as compared to healthy controls. Moreover, HD patients were found to have significantly higher endocan levels when compared with their CKD counterparts. These findings are in line with other reports [16–18]. Lee et al. [16] noted that CKD patients have significantly higher serum endocan levels as compared to controls. They also noted a significant association between serum endocan levels and poor renal function. The study of Pawlak et al. [17] found similar results. They also noted significant correlations between endocan levels and other markers including soluble intercellular (sICAM-1) and vascular cellular (sVCAM-1) adhesion molecules and tumor necrosis factor alpha (TNF-*α*), highlighting the contribution of endocan to vascular pathology and inflammation. Likewise, the study of Samouilidou et al. [18] found significantly higher endocan levels in CKD patients. Furthermore, they reported inverse correlation between endocan levels and paraoxonase 1 (PON), a marker known for its antiatherogenic properties. Their conclusions again confirm the detrimental influence of enhanced endocan expression on vascular health. Yilmaz et al. [19], in addition, found a positive correlation between endocan and CKD stage and negative correlation between it and eGFR. They also found a highly significant positive correlation between endocan and proteinuria. Similarly, Su et al. [20] found that endocan showed a drift of elevation in late-stage CKD.

In our work, HD patients with SCA had significantly higher endocan level. This novel finding is supported by similar findings reported in other patient groups. In SLE patients, Icli et al. [11] showed an association between serum endocan levels and SCA. Likewise, Lv et al. [10] found that elevated endocan levels are significant risk factors for SCA associated with diabetes mellitus. Similarly, SCA was related to serum endocan levels in psoriatic patients [12].

In our work, HD patients with SCA had significantly higher hsCRP when compared with patients without. The relation between SCA and elevated CRP levels in HD patients is well documented. Yilmaz et al. [19], Buyukhatipoglu et al. [21], and Tirmenstajn-Jankovic and Dimkovic [20] identified a relation between CRP levels and CIMT.

Interestingly, the present study identified a significant direct correlation between serum endocan and PTH levels in HD patients. The association between endocan and PTH levels was previously reported in renal transplant patients [22]. The association between endocan and PTH is probably related to the contribution of both biomarkers to the augmented inflammatory condition during HD. In our study, endocan was well correlated with the inflammatory biomarker CRP while previous reports documented the relation between PTH and CRP [23, 24].

There are multiple mechanisms that can explain the contribution of endocan to SCA in HD patients. Endocan was reportedly involved in endothelial dysfunction in many pathological conditions including sarcoidosis [25], preeclampsia [26], and silent brain infarction [27]. Endocan probably exerts its effects on the vascular endothelium through alteration of nitric oxide secretion by activation of AKT/eNOS and NF-*κ*B/iNOS pathways [28].

Moreover, endocan expression was pronounced in association with exaggerated inflammatory conditions in multiple diseases including prediabetes and type 2 diabetes [29], systemic sclerosis [30], and chronic kidney disease [31]. Gaudet et al. [32] suggested that the inflammatory actions of endocan may be mediated through control of human leukocyte trans-endothelial migration.

In conclusion, the present study suggests that serum endocan levels are related to SCA in HD patients. In addition, it is associated with the hyperinflammatory state and secondary hyperparathyroidism in those patients. Findings of this study may have therapeutic implications. Targeting endocan can be proposed as a novel approach for management of cardiovascular pathology in HD patients.

## Figures and Tables

**Table 1:** Clinical and laboratory data in the studied groups.

	HD *N* = 30	CKD *N* = 30	*p* value
Age (years) mean ± SD	55.5 ± 11.6	56.9 ± 10.5	0.62
Male/female n	20/10	16/14	0.43
*Cause of renal impairment n (%)*			
HTN	22 (90.0)	19 (63.3)	0.41
DM	13 (43.3)	15 (50.0)	0.61
PKD	1 (3.3)	—	0.31
Unknown	1 (3.3)	—	0.31
BMI (kg/m^2^) mean ± SD	28.4 ± 2.6	27.9 ± 3.7	0.52
*Laboratory findings mean* *±* *SD/median (IQR)*			
Urea (mg/dl)	163.5 (136.3–193.5)	118.5 (85.3–160.8)	*0.005*
Creatinine (mg/dl)	9.8 (7.9–10.8)	3.2 (2.2–4.8)	*<0.001*
Ca (mg/dl)	8.9 ± 1.0	8.5 ± 1.3	0.19
Po4 (mg/dl)	4.9 ± 1.8	5.5 ± 1.6	0.19
Albumin (mg/dl)	3.4 ± 0.7	4.1 ± 0.3	*<0.001*
PTH (pg/ml)	538.0 (342.3–654.8)	110.0 (50.0–170.0)	0.052
Cholesterol (mg/dl)	193.3 ± 43.6	174.1 ± 52.4	0.13
Triglycerides (mg/dl)	175.2 ± 75.1	157.3 ± 73.4	0.36
WBCs (×10^3^/ml)	6.5 ± 2.4	7.6 ± 2.5	0.1
Hb (gm/dl)	10.8 ± 1.5	10.4 ± 1.5	0.38
Platelets (×10^3^/ml)	205.2 ± 76.0	272.7 ± 86.5	*0.002*
Uric acid (mg/dl)	7.8 ± 1.5	6.2 ± 1.8	*0.001*
hsCRP (mg/dl)	28.5 (21.8–39.5)	15.0 (9.8–20.3)	*<0.001*
CIMT mm	0.80 (0.80–0.90)	0.75 (0.73–0.75)	*<0.001*
SCA *n* (%)	14 (46.7)	4 (13.3)	*0.005*
Endocan (ng/L)	519.0 (202.3–742.0)	409.0 (245.3–505.3)	*0.014*

BMI: body mass index, CIMT: carotid intima-media thickness, DM: diabetes mellitus, Hb: hemoglobin, hsCRP: high-sensitivity C-reactive protein, HTN: hypertension, PTH: parathyroid hormone, SCA: subclinical atherosclerosis, and WBCs: white blood cells. The values in italics are significant results.

**Table 2:** Comparison between HD patients with and without SCA regarding clinical and laboratory data.

	SCA + ve *n* = 14	SCA −ve *n* = 16	*p* value
Age (years) mean ± SD	60.8 ± 6.6	50.9 ± 13.2	*0.017*
Male/female n	11/3	9/7	0.2

*Cause of renal impairment n (%)*			
HTN	12 (85.7)	10 (62.5)	0.15
DM	7 (50.0)	6 (37.5)	0.49
PKD	—	1 (6.3)	0.34
Unknown	1 (7.1)	—	0.28
BMI (kg/m^2^) mean ± SD	28.1 ± 2.3	28.7 ± 2.8	0.57

*Laboratory findings mean* *±* *SD/median (IQR)*			
Urea (mg/dl)	165.0 (102.5–184.8)	162.0 (141.0–202.5)	0.58
Creatinine (mg/dl)	10.2 (7.3–11.0)	9.8 (8.2–10.7)	0.73
Ca (mg/dl)	9.4 ± 0.9	8.6 ± 1.0	*0.022*
Po4 (mg/dl)	4.1 ± 2.0	5.6 ± 1.5	*0.029*
Albumin (mg/dl)	3.6 ± 0.6	3.3 ± 0.7	0.24
PTH (pg/ml)	614.5 (571.5–752.5)	384.5 (206.5–507.3)	*0.01*
Cholesterol (mg/dl)	202.9 ± 52.1	184.9 ± 34.1	0.27
Triglycerides (mg/dl)	187.1 ± 77.7	164.8 ± 73.7	0.43
WBCs (×10^3^/ml)	6.1 ± 1.8	6.9 ± 2.8	0.35
Hb (gm/dl)	10.6 ± 1.5	10.9 ± 1.5	0.5
Platelets (×10^3^/ml)	219.3 ± 95.2	193.0 ± 54.6	0.35
Uric acid (mg/dl)	8.2 ± 1.3	7.4 ± 1.5	0.14
hsCRP (mg/dl)	36.5 (26.8–43.5)	24.0 (15.8–29.0)	*0.003*
Endocan (ng/L)	697.0 (528.3–974.8)	222.5 (158.8–565.8)	*<0.001*

The values in italics are significant results.

**Table 3:** Correlations between serum endocan levels and clinical and laboratory data in the studied patients.

	HD		CKD	
	r	*p* value	r	*p* value
Age	0.33	0.07	0.49	*0.006*
BMI	0.05	0.81	−0.26	0.17
Urea	−0.02	0.9	0.13	0.5
Creatinine	0.09	0.65	0.32	0.08
Ca	0.29	0.12	−0.13	0.49
Po4	−0.3	0.1	0.05	0.78
Albumin	−0.2	0.3	−0.16	0.39
PTH	0.5	*0.005*	−0.004	0.98
Cholesterol	0.28	0.14	−0.04	0.82
Triglycerides	0.31	0.1	0.09	0.64
WBCs	0.12	0.53	−0.06	0.74
Hb	−0.29	0.12	0.19	0.31
Platelets	0.3	0.11	−0.19	0.33
Uric acid	−0.03	0.88	0.07	0.71
hsCRP	0.55	*0.002*	0.1	0.6
CIMT	0.59	*0.001*	0.36	*0.049*

The values in italics are significant results.

## Data Availability

The data used to support the findings of this study are available from the corresponding author upon request.

## References

[1] Chen T. K., Knicely D. H., Grams M. E. (2019). Chronic kidney disease diagnosis and management: a review. *JAMA*.

[2] Jha V., Garcia-Garcia G., Iseki K. (2013). Chronic kidney disease: global dimension and perspectives. *The Lancet*.

[3] Kaminska J., Stopinski M., Mucha K. (2021). Circulating osteoprotegerin in chronic kidney disease and all-cause mortality. *International Journal of General Medicine*.

[4] Di Lullo L., House A., Gorini A., Santoboni A., Russo D., Ronco C. (2015). Chronic kidney disease and cardiovascular complications. *Heart Failure Reviews*.

[5] Kon V., Linton M. F., Fazio S. (2011). Atherosclerosis in chronic kidney disease: the role of macrophages. *Nature Reviews Nephrology*.

[6] Matsushita K., Sang Y., Ballew S. H. (2015). Subclinical atherosclerosis measures for cardiovascular prediction in CKD. *Journal of the American Society of Nephrology*.

[7] Chhajed N., Subhash Chandra B. J., Shetty M. S., Shetty C. (2014). Correlation of carotid intimal-medial thickness with estimated glomerular filtration rate and cardiovascular risk factors in chronic kidney disease. *Saudi J Kidney Dis Transpl*.

[8] Balta S., Mikhailidis D. P., Demirkol S., Ozturk C., Celik T., Iyisoy A. (2015). Endocan: a novel inflammatory indicator in cardiovascular disease?. *Atherosclerosis*.

[9] Ekiz-Bilir B., Bilir B., Aydin M., Soysal-Atile N. (2019). Evaluation of endocan and endoglin levels in chronic kidney disease due to diabetes mellitus. *Archives of Medical Science*.

[10] Lv Y., Zhang Y., Shi W. (2017). The association between endocan levels and subclinical atherosclerosis in patients with type 2 diabetes mellitus. *The American Journal of the Medical Sciences*.

[11] Icli A., Cure E., Cure M. C. (2016). Endocan levels and subclinical atherosclerosis in patients with systemic lupus erythematosus. *Angiology*.

[12] Elkamshoushi A. M., Omar S. S., El Abd A. M., Hassan S. Z., Sultan E. A., Abd Elkawy E. (2019). Subclinical atherosclerosis in psoriatic disease: relation to endocan, TNF-alpha, age of onset, and body fat. *International Journal of Dermatology*.

[13] Mulumba C., Lebughe P., Mbuyi-Muamba J. M. (2019). Prevalence and associated factors of subclinical atherosclerosis in rheumatoid arthritis at the university hospital of Kinshasa. *BMC Rheumatol*.

[14] Alay H., Laloglu E. (2022). Can endocan Be a novel marker in the diagnosis of brucellosis?. *Vector Borne and Zoonotic Diseases*.

[15] Laloglu E., Alay H. (2022). Endocan as a potential marker in diagnosis and predicting disease severity in COVID-19 patients: a promising biomarker for patients with false-negative RT-PCR. *Upsala Journal of Medical Sciences*.

[16] Lee Y. H., Kim J. S., Kim S. Y. (2016). Plasma endocan level and prognosis of immunoglobulin A nephropathy. *Kidney Research and Clinical Practice*.

[17] Pawlak K., Mysliwiec M., Pawlak D. (2015). Endocan--the new endothelial activation marker independently associated with soluble endothelial adhesion molecules in uraemic patients with cardiovascular disease. *Clinical Biochemistry*.

[18] Samouilidou E., Bountou E., Papandroulaki F., Papamanolis M., Papakostas D., Grapsa E. (2018). Serum endocan levels are associated with paraoxonase 1 concentration in patients with chronic kidney disease. *Therapeutic Apheresis and Dialysis*.

[19] Yilmaz G., Ustundag S., Temizoz O. (2015). Fibroblast growth factor-23 and carotid artery intima media thickness in chronic kidney disease. *Clinical Laboratory*.

[20] Tirmenstajn-Jankovic B., Dimkovic N. (2005). C-reactive protein as an independent risk factor for carotid atherosclerosis in hemodialysis patients. *Medicinski Pregled*.

[21] Buyukhatipoglu H., Tiryaki O., Tahta K., Usalan C. (2007). Inflammation as a risk factor for carotid intimal-medial thickening, a measure of subclinical atherosclerosis in haemodialysis patients: the role of chlamydia and cytomegalovirus infection. *Nephrology*.

[22] Atis O., Keles M., Cankaya E., Dogan H., Aksoy H., Akcay F. (2016). Vitamin D treatment effect on serum endocan and high-sensitivity C-reactive protein levels in renal transplant patients. *Progress in Transplantation*.

[23] Jean G., Souberbielle J. C., Zaoui E. (2016). Analysis of the kinetics of the parathyroid hormone, and of associated patient outcomes, in a cohort of haemodialysis patients. *BMC Nephrology*.

[24] Abdel-Messeih P. L., Alkady M. M., Nosseir N. M., Tawfik M. (2020 Oct 2). Inflammatory markers in end-stage renal disease patients on haemodialysis. *Journal of Medical Biochemistry*.

[25] Aciksari G., Kavas M., Atici A. (2018). Endocan levels and endothelial dysfunction in patients with sarcoidosis. *Angiology*.

[26] Cross S. N., Buhimschi I. A., Buniak C. D. (2022). Endocan, a soluble marker of endothelial cell activation is a molecular marker of disease severity in women with preeclampsia. *Reproductive Sciences*.

[27] Erdal Y., Yavuz N., Oguz O., Mahmutoglu A. S., Emre U. (2021). Endocan: a novel predictor of endothelial dysfunction in silent brain infarction. *Angiology*.

[28] Kumar S. K., Mani K. P. (2021). Endocan alters nitric oxide production in endothelial cells by targeting AKT/eNOS and NFkB/iNOS signaling. *Nitric Oxide*.

[29] Klisic A., Kavaric N., Stanisic V. (2020). Endocan and a novel score for dyslipidemia, oxidative stress and inflammation (DOI score) are independently correlated with glycated hemoglobin (HbA1c) in patients with prediabetes and type 2 diabetes. *Archives of Medical Science*.

[30] Lo Gullo A., Mandraffino G., Rodriguez-Carrio J. (2021). Endocan and circulating progenitor cells in women with systemic sclerosis: association with inflammation and pulmonary hypertension. *Biomedicines*.

[31] Yilmaz M. I., Siriopol D., Saglam M. (2014). Plasma endocan levels associate with inflammation, vascular abnormalities, cardiovascular events, and survival in chronic kidney disease. *Kidney International*.

[32] Gaudet A., Portier L., Mathieu D. (2020). Cleaved endocan acts as a biologic competitor of endocan in the control of ICAM-1-dependent leukocyte diapedesis. *Journal of Leukocyte Biology*.

